# Closed-wound negative pressure therapy dressing after loop ostomy closure: a retrospective comparative study

**DOI:** 10.1038/s41598-022-11856-8

**Published:** 2022-05-12

**Authors:** P. Curchod, D. Clerc, J. Jurt, M. Hubner, D. Hahnloser, N. Demartines, F. Grass

**Affiliations:** Department of Visceral Surgery, Lausanne University Hospital (CHUV), University of Lausanne (UNIL), 26, Rue du Bugnon, 1010 Lausanne, Suisse

**Keywords:** Functional gastrointestinal disorders, Gastrointestinal cancer, Inflammatory bowel disease, Gastrointestinal cancer, Colorectal cancer

## Abstract

Closed-wound negative pressure wound therapy (NPWT) dressings were recently introduced with the purpose to reduce incisional surgical site infections (iSSI) in high-risk wounds. The aim of this study was to compare iSSI rates in patients after ostomy closure with and without additional application of a closed-wound NPWT dressing. Single-center retrospective analysis of consecutive patients undergoing ileo- or colostomy closure over an 8-year period (January 2013—January 2021). Intradermal non-purse string technique with absorbable sutures were used in all patients. Since November 2018, all patients (study group) received a NPWT device for a maximum of 5 days postoperatively (PICO, SMITH AND NEPHEW). Primary outcome was iSSI rate within 30 days of surgery. SSI was defined in accordance with the Center of Disease Control (CDC) classification and included superficial and deep incisional SSI. Data was retrieved from the institutional enhanced recovery after surgery (ERAS) database, with standardized complication assessment by trained abstractors. In total, 85 patients (25%) in the study group were comparable with 252 (75%) patients in the control group regarding demographics (age, gender, body mass index, ASA score), ostomy type and anastomotic technique (all *p* > 0.05), but not wound contamination class (class III: 5% vs 0%, *p* < 0.001). Median time to NPWT removal was 4 (IQR 3–5) days. Incisional SSI were observed in 4 patients (4.7%) in the study group and in 27 patients (10.7%) in the control group (*p* = 0.097). These preliminary results suggest a potential benefit of systematic application of the NPWT device after loop ostomy closure. A randomized controlled study is needed.

## Introduction

Incisional surgical site infection (iSSI) is the most common complication after stoma closure, with reported rates varying from 2 to 41%^1,2^.

Among various skin closure techniques for ostomy wounds^3^, purse-string closure has shown superiority in reducing SSI compared to primary skin closure^4–6^. Nevertheless, purse-string wounds take longer to heal than primary closure techniques and require daily wound care^4^.

Closed-wound negative pressure wound therapy (NPWT) has been suggested as a iSSI reducing alternative in high-risk wounds^7^. However, data on closed-wound NPWT application after loop ostomy closure are scarce, despite encouraging preliminary results regarding clinical benefits and ease of use^8–10^.

The aim of this study was to compare iSSI rates in patients after loop ostomy closure with primary skin closure with or without application of a closed-wound NPWT dressing.

## Methods

Retrospective comparative study of consecutive patients undergoing loop ileostomy or colostomy closure between January 2013 and January 2021, conducted at Lausanne University Hospital (CHUV). Since January 2013, all patients were treated within an Enhanced Recovery After Surgery (ERAS) pathway and data were prospectively collected in a dedicated ERAS database. A new dressing protocol including the NPWT device was implemented systematically in November 2018 for all patients undergoing ostomy closure. These patients (study group) were compared with the cohort operated before this date (control group). The present study was approved by the ethics committee of Canton de Vaud (*Commission cantonale d’éthique de la recherche sur l’être humain*—CER-VD). Due to retrospective nature of the study informed consent was waived by the ethics committee (decision # 2020–238). All methods were performed in accordance with the relevant guidelines and regulations.

Baseline demographics included age, gender, body mass index (BMI), American Society of Anesthesiologists (ASA) score, current smoking status, pre-existing diabetes requiring medication, ongoing systemic chemotherapy (within 3 months before surgery) and immunosuppressive medication (within two weeks prior to surgery). Immunosuppressive medications included systemic steroid therapy (> 10 mg/day) and specific immunosuppressive drugs. Intra-operative details such as wound contamination class (defined according to the CDC classification)^11^, type of anastomosis and duration of surgery were collected.

All procedures were elective loop ileostomy or colostomy closures through direct approach. All procedures were carried out by board-certified general or colorectal surgeons. Patients not directly operated through the existing stoma site or undergoing additional abdominal procedures were excluded.

### Wound management

**Figure 1:**
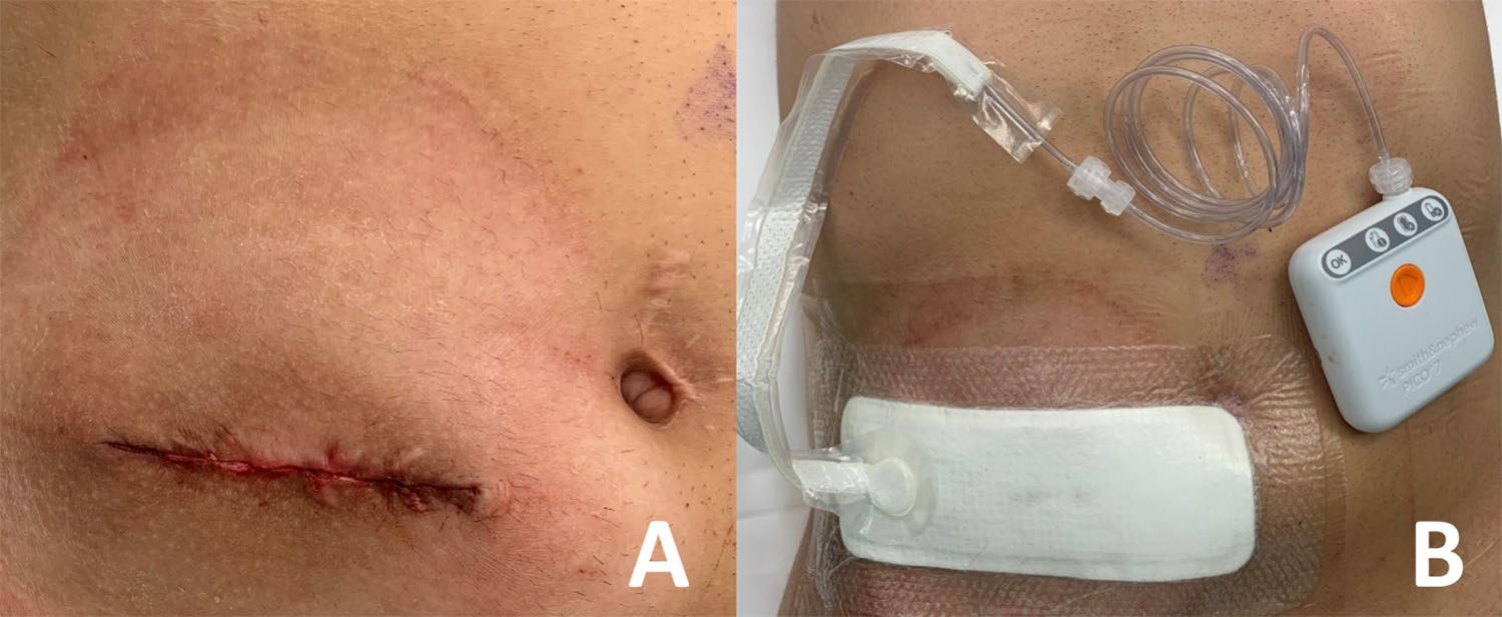
Wound management. Figure depicting wound management after ostomy closure. (**A**) Direct skin closure with intradermal absorbable suture. (**B**) Application of single-use closed-wound negative pressure wound therapy device.

Ostomy wounds were closed plane-by-plane in a standardized fashion. The fascia was closed with separate stitches of monofilament synthetic absorbable sutures (PDS-1, ETHICON INC, Raritan, USA) and subcutaneous tissue was adapted through separate stitches of synthetic absorbable braided sutures (Vicryl, ETHICON INC, Raritan, USA). The cutaneous layer was closed with intradermal absorbable sutures (Monocryl 4.0, ETHICON INC, Raritan, USA) in a linear fashion, according to institutional protocol (Fig. 1A). Wound dressings included surgical glue (Histoacryl, B BRAUN INC, Sempach, CH) for all patients in the control group. Since November 2018, a single-use closed-wound negative pressure wound therapy (NPWT) device (PICO, SMITH AND NEPHEW INC, Hull, UK) was applied systematically in all patients instead of surgical glue as part of an institutional SSI prevention quality improvement initiative (NPWT group) (Fig. 1B). The NPWT device was left in place until post-operative day 5 or the day of discharge if earlier.

### Outcomes

Primary outcome was iSSI within 30 days post-operatively. SSI was defined in accordance with the CDC classification^11^, with iSSI regrouping superficial (CDC—A) and deep (CDC—B) incisional SSI. Data was retrieved from the institutional enhanced recovery after surgery (ERAS) database, with standardized complication assessment by trained abstractors for entire study period. An appointment was scheduled 6 weeks after surgery. Suggesting that there is a 30 days follow up for all patients. Secondary outcomes were length of hospital stay (LOS), return to operating room (OR) and postoperative complications at 30 days, classified according to the validated Clavien classification^12^, with grade ≥ 3b defining major morbidity. Anastomotic leak was defined as a clinically symptomatic leak requiring return to OR.

### Statistical analysis

Continuous variables were summarized as median (interquartile range: IQR) or mean ± standard deviation (SD) and categorical variables as frequencies and percentages.

Chi-square and 'Student's t-test were used for comparison of categorical and continuous variables, respectively. Statistical significance was defined as *p* ≤ 0.05. Both matching (similarity of baseline characteristics in both comparative groups) and multivariable analysis (low event rate) were not performed.

### Conference presentation

The content of this manuscript was presented at the Annual Meeting of the Swiss Society of Surgery on 3 June 2021.

## Results

In total, 337 patients were included. The NPWT group (study group) included 85 patients (25%), while 252 patients (75%) had conventional primary skin closure (control group). Baseline demographics were comparable between both groups (all *p* > 0.05, Table 1).

**Table 1:** Demographics and surgical details.

	Control group n = 252	NPWT group n = 85	Total n = 337	P value
Age (mean ± SD)	59 ± 16	58 ± 17	59 ± 17	0.492
Male gender (%)	146 (57.9)	51 (60.0)	197 (58.4)	0.800
ASA class ≥ 3 (%)	55 (21.8)	27 (31.7)	82 (24.3)	0.079
BMI (kg/m^2^, mean ± SD)	24.7 ± 5.2	25.8 ± 5	25 ± 5.2	0.069
Smoker (%)	60 (23.8)	21 (24.7)	81 (24.0)	0.884
Diabetes (%)	22 (8.7)	5 (5.8)	27 (8.0)	0.494
Chemotherapy (%)	34 (13.5)	15 (17.6)	49 (14.5)	0.375
Immunosuppression (%)	18 (7.14)	2 (2.3)	20 (5.9)	0.120
Ileostomy:Colostomy ratio	217:35	68:17	285:52	0.223
Handsewn anastomosis (%)	217 (86.1)	66 (77.6)	283 (84.0)	0.076
Contamination class (%)				** < 0.001**
II	252 (100)	80 (94.1)	332 (98.5)	
III	0	5 (5.8)	5 (1.4)	

Baseline demographic parameters of patients before and after implementation of NPWT wound device.

SD—standard deviation, ASA—American Society of Anaesthesiology, BMI—body mass index.

Age and BMI are presented as mean ± standard deviation (SD). All others are frequency with percentage.

Bold P values indicate statistical significance (*p* < 0.05).

Surgical details are depicted in Table 1. The proportion of ileostomy and colostomy closure was similar in both groups (*p* = 0.22). The majority of anastomoses were handsewn in both groups (overall 84%). Wound contamination class II (clean-contaminated) was reported in 94% of patients in the NPWT group, compared to 100% in the control group. Median surgical duration was around 10 min longer in the NPWT group (*p* = 0.004).

Postoperative outcomes are detailed in Table 2.

**Table 2:** Postoperative outcomes.

	Control group n = 252	NPWT group n = 85	Total n = 337	P value
Incisional SSI (%)	27 (10.7)	4 (4.7)	31 (9.1)	0.097
Organ/space SSI (%)	8 (3.1)	1 (1.1)	9 (2.6)	0.323
Any complication (%)	109 (43.2)	29 (34.1)	138 (40.9)	0.200
Major (%)	21 (8.3)	9 (10.6)	29 (8.6)	0.528
Return to OR (%)	15 (5.9)	5 (5.8)	20 (5.9)	0.981
Anastomotic leak (%)	5 (1.9)	1 (1.1)	6 (1.7)	0.626
LOS (days, median, IQR)	4 (3, 6)	4 (3, 6)	4 (3, 6)	0.058
NPWT treatment duration (days, median, IQR)	N/A	4 (3, 5)	N/A	–
Device malfunction (%)	N/A	2 (2.3)	N/A	–

Primary and secondary outcomes of patients before and after implementation of NPWT wound device.

SSI—surgical site infection, OR—operating room, LOS-Length of stay, IQR—Interquartile range, NPWT—negative pressure wound therapy.

Median time to NPWT removal was 4 days (IQR 3–5). Device malfunction was observed in two patients (2.3%), requiring early removal of the NPWT dressing. Median hospital stay was 4 (IQR 3–6) days in both groups.

Incisional surgical site infections were observed in 4 patients (4.7%) in the NPWT group and in 27 patients (10.7%) in the control group (*p* = 0.097). Overall postoperative morbidity, major complications, return to OR and anastomotic leak rates were all similar in both groups (Table 2). Overall, one patient died in the control group due to malignant arrhythmia at POD 8.

## Discussion

The present study suggests a potential benefit associated with the systematic application of a closed-wound NPWT device after loop ostomy closure. Technical issues were very uncommon. Adequate powered randomized trials are needed to confirm these preliminary findings.

NPWT has been established as a SSI preventing measure for several different indications, especially in colorectal surgery^7^. However, the NEPTUNE trial did not describe NPWT as superior to classical wound closure^13^.

Nevertheless, a few studies assessed the effect of NPWT application after loop ostomy closure^8–10^. A recently published randomized controlled study of NPWT in primarily closed wounds after loop ileostomy closure described a iSSI rate of 5.7% in the NPWT group vs. 19% without NPWT^9^. The present series revealed a similar rate of iSSI in the NPWT group, but lower rates in the control group, which may explain the lack of significant difference in the present comparison. However, the low SSI rate may also be related to underreporting as a potential bias of retrospective studies. A recent observational study from Japan evaluating an open-wound VAC therapy system customized for closed wounds did not report any SSI in 50 patients after ileostomy closure^10^. While their results were promising, the customized technique of NPWT application on open wounds may be difficult to apply on a large scale.

In this present series, NPWT dressings were well-tolerated and easy to use, with only exceptional technical issues. Surgical duration was slightly longer, which however may also be related to constitutional factors (higher BMI and comorbidity indices in the NPWT group). The main advantage probably consists in simplified post-operative wound care, unlike the time-consuming purse-string closure. While several reports revealed lower SSI rates after purse-string closure compared to primary closure^4,5^, wounds require daily care until discharge and specialist wound care for about 35 days according to our institutional experience (4) and a previous report^14^. In contrast, primarily closed ostomy wounds without SSI occurrence typically heal within 7–24 days^4,9^. The intradermal suture is further convenient since no follow-up care is needed after device removal providing uneventful wound healing.

The present study has several limitations related to its retrospective nature without dedicated preset defined data, the small sample size of the study group and the uncontrolled setting. However, consecutive patients were included to limit any potential selection bias. Based on the positive preliminary experience of other series, our group decided to implement closed-wound NPWT therapy as a new standard of care (practice change) and to compare outcomes to the unselected pre-implementation cohort. This design was chosen given the consistency of surgical and perioperative care and standardized, prospective SSI surveillance based on our institutional ERAS protocol^15^.

An adequately powered randomized controlled multicentric trial comparing different techniques and considering patient preference might probably be the most appropriate way to further optimize ostomy wound management. The ongoing SR-PICO randomized study (KCT0004063) may confirm our preliminary results^16^.

In conclusion, additional closed-wound NPWT dressings after primary skin closure of ostomy wounds seems beneficial in reducing iSSI. This strategy challenges the purse-string closure method in ease of management, reduction of resources and time to complete wound healing.

## Abbreviations

ASA: American society of anesthesiologists
BMI: Body mass index
CDC: Center of disease control
CHUV: Centre hospitalier universitaire vaudois
ERAS: Enhanced recovery after surgery
IQR: Interquartile range
iSSI: Incisional surgical site infection
LOS: Length of stay
OR: Operating room
NPWT: Negative pressure wound therapy
POD: Post-operative day
SD: Standard deviation
SSI: Surgical site infection
VAC: Vacuum-assisted closure

## References

[1] de Paula TR, Nemeth S, Kiran RP, Keller DS (2020). Predictors of complications from stoma closure in elective colorectal surgery: an assessment from the American College of Surgeons National Surgical Quality Improvement Program (ACSNSQIP). Tech. Coloproctol..

[2] Marquez TT, Christoforidis D, Abraham A, Madoff RD, Rothenberger DA (2010). Wound infection following stoma takedown: primary skin closure versus subcuticular purse-string suture. World J. Surg..

[3] Li LT, Brahmbhatt R, Hicks SC, Davila JA, Berger DH, Liang MK (2014). Prevalence of surgical site infection at the stoma site following four skin closure techniques: a retrospective cohort study. Dig. Surg..

[4] Lee JT, Marquez TT, Clerc D, Gie O, Demartines N, Madoff RD (2014). Pursestring closure of the stoma site leads to fewer wound infections: results from a multicenter randomized controlled trial. Dis. Colon Rectum..

[5] Hajibandeh S, Hajibandeh S, Kennedy-Dalby A, Rehman S, Zadeh RA (2018). Purse-string skin closure versus linear skin closure techniques in stoma closure: a comprehensive meta-analysis with trial sequential analysis of randomised trials. Int. J. Colorectal. Dis..

[6] McCartan DP, Burke JP, Walsh SR, Coffey JC (2013). Purse-string approximation is superior to primary skin closure following stoma reversal: a systematic review and meta-analysis. Tech. Coloproctol..

[7] Sahebally SM, McKevitt K, Stephens I, Fitzpatrick F, Deasy J, Burke JP (2018). Negative pressure wound therapy for closed laparotomy incisions in general and colorectal surgery: a systematic review and meta-analysis. JAMA Surg..

[8] Obeid N, Sharma E, Dunstan M, Nisar P, Trivedi P, Madani R (2021). Negative pressure therapy for stoma closure sites-a nonrandomised case control study. Int. J. Colorectal. Dis..

[9] Wierdak M, Pisarska-Adamczyk M, Wysocki M, Major P, Kolodziejska K, Nowakowski M (2021). Prophylactic negative-pressure wound therapy after ileostomy reversal for the prevention of wound healing complications in colorectal cancer patients: a randomized controlled trial. Tech. Coloproctol..

[10] Okuya K, Takemasa I, Tsuruma T, Noda A, Sasaki K, Ueki T (2020). Evaluation of negative-pressure wound therapy for surgical site infections after ileostomy closure in colorectal cancer patients: a prospective multicenter study. Surg. Today..

[11] Horan TC, Gaynes RP, Martone WJ, Jarvis WR, Emori TG (1992). CDC definitions of nosocomial surgical site infections, 1992: a modification of CDC definitions of surgical wound infections. Am. J. Infect. Control..

[12] Dindo D, Demartines N, Clavien PA (2004). Classification of surgical complications: a new proposal with evaluation in a cohort of 6336 patients and results of a survey. Ann. Surg..

[13] Murphy PB, Knowles S, Chadi SA, Vogt K, Brackstone M, Koughnett JAV (2019). Negative pressure wound therapy use to decrease surgical nosocomial events in colorectal resections (NEPTUNE): a randomized controlled trial. Ann. Surg..

[14] Camacho-Mauries D, Rodriguez-Diaz JL, Salgado-Nesme N, Gonzalez QH, Vergara-Fernandez O (2013). Randomized clinical trial of intestinal ostomy takedown comparing pursestring wound closure vs conventional closure to eliminate the risk of wound infection. Dis. Colon. Rectum..

[15] Slieker J, Hubner M, Addor V, Duvoisin C, Demartines N, Hahnloser D (2018). Application of an enhanced recovery pathway for ileostomy closure: a case-control trial with surprising results. Tech. Coloproctol..

[16] Kim S, Kang SI (2020). The effectiveness of negative-pressure wound therapy for wound healing after stoma reversal: a randomised control study (SR-PICO study). Trials.

